# Unusual Anastomosis between Branches of the Ulnar Nerve (Kaplan Type Anastomotic Variant) – Case Report

**DOI:** 10.1055/s-0042-1744295

**Published:** 2022-06-10

**Authors:** Kennedy Martinez Oliveira, Gabriel das Chagas Benevenuto, Ana Clara Ferreira de Almeida, Daniel Gonçalves de Oliveira, Leonardo Braga Bueno, Maria Fernanda Carvalho Teixeira

**Affiliations:** 1 Departamento de Anatomia e Imagem (IMA), Faculdade de Medicina, Universidade Federal de Minas Gerais (UFMG), Belo Horizonte, MG, Brasil; 2 Departamento de Ciências Básicas da Vida (DCBV), Faculdade de Medicina, Universidade Federal de Juiz de Fora (UFJF), Juiz de Fora, MG, Brasil

**Keywords:** anatomy, cadaver, hand/surgery, ulnar nerve

## Abstract

The Kaplan anastomosis is a rare communication originally described between the superficial and dorsal branches of the ulnar nerve, distal to the ulnar tunnel, and in strict relation with the pisiform bone. It reveals, by its particular location, a formation of high clinical-surgical expressiveness. In this paper, we describe a Kaplan-type communication from a left upper limb with an unusual loop conformation between branches of the ulnar nerve in the pisiform bone.

## Introduction

The ulnar nerve is vulnerable due to its extensive and complex topographic path, along with formations such as the Struther arcade, the proximal ulnar tunnel, the retrocondylar retinaculum (the Osborne ligament), the aponeurosis of the flexor carpi ulnaris muscle (the Osborne fascia), the hiatus of the fascial sheath of the flexor digitorum superficialis muscle (the Spinner ligament) and the distal ulnar tunnel (the Guyon canal), with possibilities of neuropathic occurrences by compression. In association with these osteofibrous and/or musculofascial complexities, many of which are atavistic, the ulnar nerve presents anastomoses of relevant clinical-surgical and phylogenetic significance: Martin-Gruber, Marinacci, Riche-Cannieu, and Berrettini. 1 Besides these, there are the variable anastomosis or communications between branches of the ulnar nerve, such as the rare anastomosis between the dorsal branch and the proper (medial) digital branch of the V finger described by Kaplan in 1963. 2 3 Thus, we report an unusual Kaplan-type anastomosis in which one of the divisions of the dorsal branch of the ulnar nerve anastomoses completely (without emission of cutaneous branches), in a middle position between the ulnar nerve and its superficial and deep branches, and in a narrow loop in the pisiform bone.

## Case Report

This work resulted from the neurovascular dissection of a left upper limb from a male cadaver, age unknown, and preserved in 10% formaldehyde solution. The morphometric results, despite the fibroelastic contractions common to preserved tissues, were obtained using a digital pachymeter (Western Pro®) (Western, São Paulo, SP, Brasil), resolution 0.1 mm, a millimeter stainless steel Rhosse ruler (Rhosse Instrumentos e Equipamentos Cirúrgicos. Ribeirão Preto, SP, Brasil), and a dry-tipped, stainless steel Jon compass (Jon Odontologia Ltda. São Paulo, SP, Brasil). As this is a descriptive analysis on a cadaver already under the supervision of the laboratory this work does not require the approval of the Ethics Committee, according to Law 8501/92 and Resolution 196/96 of the National Health Council, followed by Provision/CG No. 16, September 26, 1997.

**Fig. 1:**
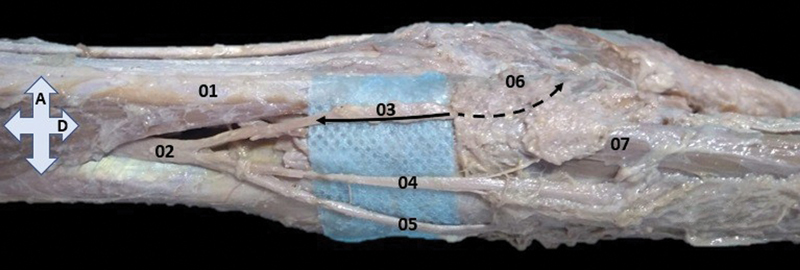
Medial view of the forearm and hand. 01–tendon of the flexor carpi ulnaris muscle; 02–dorsal branch of the ulnar nerve; 03–medial division of the dorsal branch of the ulnar nerve, the filled arrow indicates the dissected segment and the interrupted arrow indicates the path associated to the pisiform bone and with fibromuscular tissue overlay; 04–intermediary division of the dorsal branch of the ulnar nerve; 05–lateral division of the dorsal branch of the ulnar nerve; 06–pisiform bone; 07–abductor digiti minimi muscle; A–anterior, and D–distal.

**Fig. 2:**
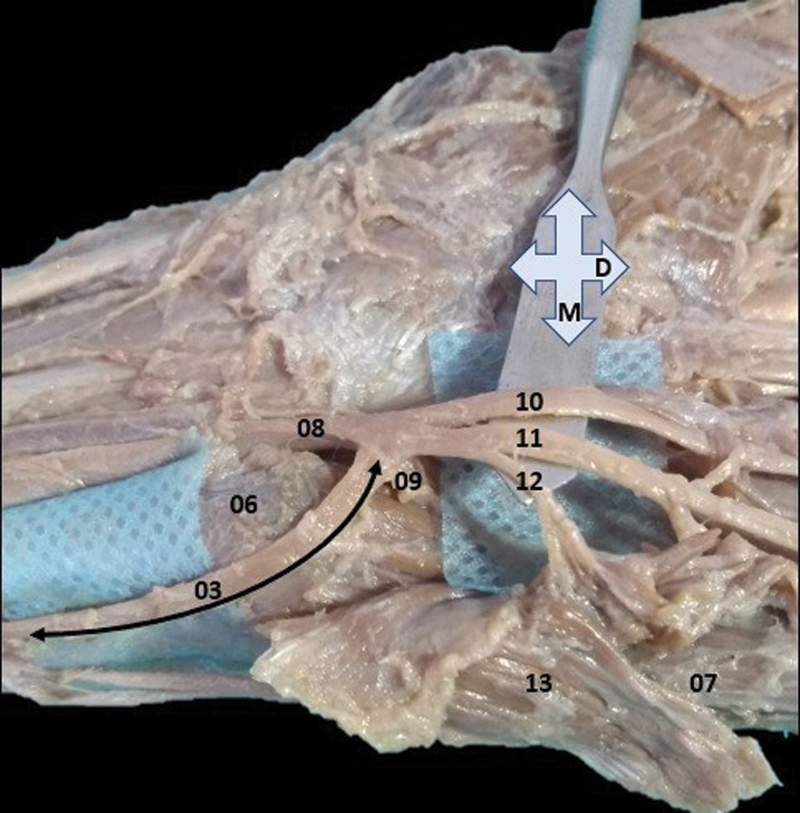
Volar view with slight displacement of the nerve structures as from the metal spatula. 03–completely dissected medial division of the dorsal branch of the ulnar nerve (double filled arrow) and in anastomosis at the midpoint between the ulnar nerve (08), the deep branch of the ulnar nerve (09) and the superficial branch of the ulnar nerve (the Kaplan anastomosis variant); 06–pisiform bone; 07–abductor digiti minimi muscle; 10–common palmar digital nerve; 11–proper palmar digital nerve; 12–motor branch for the palmaris brevis muscle; and 13–deep face of the palmaris brevis muscle, partially retracted from its origin or distal insertion M–medial and D–distal.

**Fig. 3:**
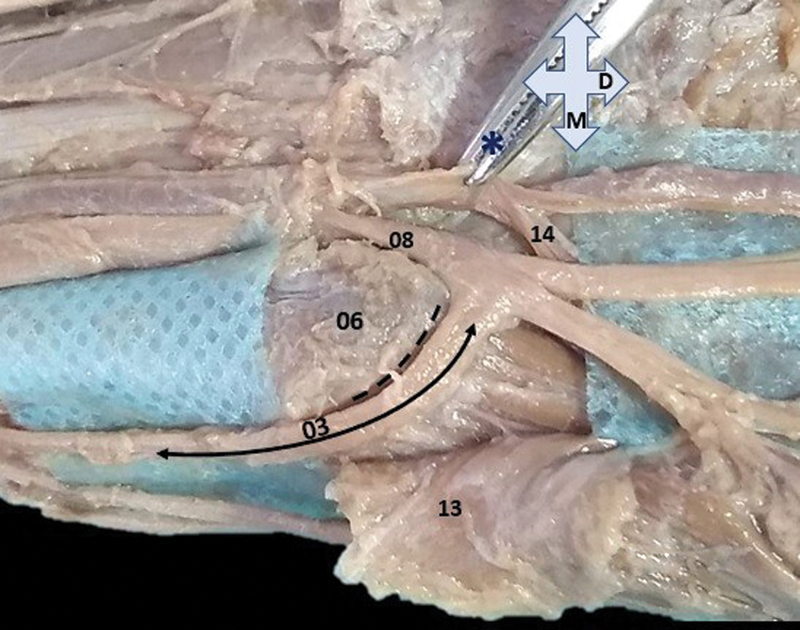
Sulcus in the pisiform bone (6) represented by the discontinuous black line in the sulcus and in the medial and anterior aspect of this bone; 03–Kaplan-type anastomosis (double filled arrow); 08–ulnar nerve; 13–deep face of the palmaris brevis muscle, partially retracted from its origin or proximal insertion; 14–deep branch of the ulnar artery; asterisk (*) – surgical instrument pulling the ulnar artery as from its adventitia; M – medial, and D – distal.

The ulnar nerve was completely dissected in its antebrachial and hand segments. The dissection revealed, from the dorsal branch of the ulnar nerve, and immediately posterior to the distal musculotendinous connection of the flexor carpi ulnaris muscle, the emission of the three branches—medial, intermedius, and lateral. The medial branch followed to the palmar aspect and parallel to the flexor carpi ulnaris muscle's tendon, causing a very prominent sulcus in the anterior and medial aspects of the pisiform bone, superficial to the origin of the abductor digiti minimi muscle. The medial branch anastomosed completely, in a loop, at the level of the pisiform bone, at the midpoint between the superficial and deep branches of the ulnar nerve ( Figs. 1 and 2 ), without muscular and/or cutaneous innervation of this branch along its course. The emission of the three branches occurred 4.75 cm from the apex of the ulnar styloid process. The intermedius and lateral branches followed in obliquity to the subcutaneous of the dorsum of the hand and fingers IV and V, forming the dorsal digital branches. The sulcus in the pisiform bone presented, in its most rugged point—in the medial or ulnar aspect, a maximum depth of 0.2 mm ( Fig. 3 ) with a general conformation of pulley or trochlea. On the lateral aspect of the pisiform bone (radial), the superficial branch of the ulnar nerve caused a continuous sulcus of lesser depth in the anteromedial direction. Additionally, the superficial branch of the ulnar nerve, after the retraction of the transverse carpal ligament and the palmaris brevis muscle, characteristically emitted the motor branch to the palmaris brevis muscle and the two digital branches—proper and common ( Fig. 2 ). However, the motor branch for the palmaris brevis muscle originated from the medial margin of the digital branch proper for the minimi finger. The palmar branch of the ulnar nerve was not preserved in this anatomical preparation. There were no vascular variations.

## Discussion

The Kaplan anastomosis constitutes a rare communication between the proper digital branch of the minimi finger (medial) and the dorsal branch of the ulnar nerve 4 5 6 7 and presents, due to its superficial location, significant clinical and surgical implications, including iatrogenic. 8 However, there are variations of this anastomosis such as the atypical connection of the dorsal branch to the deep branch of the ulnar nerve as described by Ghabriel and Makar. 9 In this study, the anastomosis occurred between the medial division of the dorsal branch of the ulnar nerve at a midpoint between the origins of the superficial and deep branches, a finding that differs from those of Paraskevas et al. 6 and Torre et al., 7 in which both studies reported anastomosis of the dorsal branch of the ulnar nerve to the ulnar nerve proximal to the emissions of the superficial and deep branches. In addition, the anastomotic medial division did not suppress the cutaneous aspect of the medial margin of the hand in direction of the dorsal face, which differs from previous papers. Hankins and Flemming, 4 proposed a classification, into six types, for variations in the Kaplan anastomosis concerning the connection and/or distal communication of the dorsal branch of the ulnar nerve. However, the present study does not fit into this classification, because there was no prevision of communication of the medial division of the dorsal branch at the midpoint between the volar branches of the ulnar nerve (superficial and deep), and because the medial division relates exclusively to the anastomosis. The findings of these authors evidenced a short sulcus in the pisiform bone for the accommodation of the variant nerve branch, which we also observed, however, the sulcus was of greater extension and depth ( Fig. 3 ).
