# Supplementary material for: Unusual Anastomosis between Branches of the Ulnar Nerve (Kaplan Type Anastomotic Variant) – Case Report
Source: Rev Bras Ortop (Sao Paulo). 2022 Jun 10;59(Suppl 1):e65–7. doi: 10.1055/s-0042-1744295 (PMC11254421; doi:10.1055/s-0042-1744295)
Supplement: Related texts

# Research article: Anastomose incomum entre ramos do nervo ulnar (variante anastomótica de Kaplan) – Relato de caso

**Keywords:** anatomia, cadáver, mão/cirurgia, nervo ulnar

## Abstract

A anastomose de Kaplan é uma rara comunicação originalmente descrita entre os ramos superficial e dorsal do nervo ulnar, distal ao túnel ulnar e em estreita relação com o osso pisiforme. O que revela, pela sua particular localização, uma formação de alta expressividade clínico-cirúrgica. Neste trabalho, uma comunicação do tipo Kaplan, porém, ainda não relatada, é descrita a partir de um membro superior esquerdo com uma inusual conformação em alça, ou um *looping* , entre ramos do nervo ulnar, no osso pisiforme.

## Introdução

O nervo ulnar apresenta-se vulnerável devido ao seu extenso e complexo trajeto topográfico junto a formações como a arcada de Struthers, o túnel ulnar, o retináculo cubital (ligamento de Osborne), a aponeurose do músculo flexor ulnar do carpo (fáscia de Osborne), o hiato da bainha fascial do músculo flexor superficial dos dedos (ligamento de Spinner) e do túnel ulnar distal (canal de Guyon), com possibilidades de ocorrências neuropáticas por compressão. Associadas a essas complexidades osteofibrosas e/ou musculofaciais, muitas delas atávicas, o nervo ulnar apresenta anastomoses expressivas no campo clínico-cirúrgico e filogenético: de Martin-Gruber, de Marinacci, de Riche-Cannieu e de Berrettini. 1 Além destas, existem as anastomoses variáveis ou conexões entre ramos do nervo ulnar, como a rara anastomose entre o ramo dorsal e o ramo digital próprio (medial) do dedo V descrita por Kaplan em 1963. 2 3 Sendo assim, relatamos uma anastomose incomum do tipo Kaplan em que uma das divisões do ramo dorsal do nervo ulnar se anastomosa completamente (sem emissão de ramos cutâneos), em posição média, entre o nervo ulnar e seus ramos superficial e profundo, e em uma alça estreita no osso pisiforme.

## Relato de Caso

Este estudo é o resultado da dissecção neurovascular do membro superior esquerdo de um cadáver do sexo masculino, de idade desconhecida, e preservado em solução de formaldeído a 10%. Os resultados morfométricos, apesar das contrações fibroelásticas comuns aos tecidos preservados, foram obtidos com paquímetro digital (Western, São Paulo, SP, Brasil), resolução 0,1 mm, régua milimetrada Rhosse de aço inoxidável (Rhosse Instrumentos e Equipamentos Cirúrgicos. Ribeirão Preto, SP, Brasil) e compasso Jon (Jon Odontologia Ltda. São Paulo, SP, Brasil) de ponta seca de aço inoxidável da marca . Por se tratar de uma análise descritiva em cadáver já sob supervisão do laboratório, este trabalho dispensa a aprovação do Comitê de Ética, conforme Lei 8.501/92 e Resolução 196/96 do Conselho Nacional de Saúde, seguida do Provimento/CG n° 16, de 26 de setembro de 1997.

O nervo ulnar foi completamente dissecado em seus segmentos antebraquial e de mão. A dissecção revelou, a partir do ramo dorsal do nervo ulnar, e imediatamente posterior à conexão musculotendínea distal do músculo flexor ulnar do carpo, a emissão dos três ramos – medial, intermediário e lateral. O ramo medial seguiu para a face palmar paralelamente ao tendão do músculo flexor ulnar do carpo, causando um sulco muito proeminente nas faces anterior e medial do osso pisiforme, superficial à origem do músculo abdutor do dedo mínimo. O ramo medial anastomosou-se completamente, em alça, ao nível do osso pisiforme, no ponto médio entre os ramos superficial e profundo do nervo ulnar ( Figs. 1 e 2 ), sem inervação muscular e/ou cutânea desse ramo ao longo de seu trajeto. A emissão dos três ramos ocorreu a 4,75 cm do ápice do processo estiloide da ulna. Os ramos intermediário e lateral seguiram em obliquidade ao subcutâneo do dorso da mão e dedos IV e V, formando os ramos digitais dorsais. O sulco do osso pisiforme apresentou, em seu ponto mais acidentado, na face medial ou ulnar, profundidade máxima de 0,2 mm ( Fig. 3 ) com conformação geral de polia ou tróclea. Na face lateral do osso pisiforme (radial), o ramo superficial do nervo ulnar causou um sulco contínuo de menor profundidade no sentido anteromedial. Além disso, o ramo superficial do nervo ulnar, após a retração do ligamento transverso do carpo e do músculo palmar curto, emitiu caracteristicamente o ramo motor para o músculo palmar curto e os dois ramos digitais – próprio e comum ( Fig. 2 ). Entretanto, o ramo motor para o músculo palmar curto originou-se da margem medial do ramo digital próprio do dedo mínimo. O ramo palmar do nervo ulnar não foi preservado neste preparo anatômico. Não houve variações vasculares.

**Fig. 1:** Vista medial do antebraço e mão. 01–tendão do músculo flexor ulnar do carpo; 02–ramo dorsal do nervo ulnar; 03–divisão medial do ramo dorsal do nervo ulnar, a seta preenchida indica o segmento dissecado e a seta interrompida indica o trajeto associado ao osso pisiforme e com sobreposição de tecido fibromuscular; 04–divisão intermediária do ramo dorsal do nervo ulnar; 05–divisão lateral do ramo dorsal do nervo ulnar; 06–osso pisiforme; 07–músculo abdutor do dedo mínimo; A–anterior e D–distal.

**Fig. 2:** Vista volar com leve deslocamento das estruturas nervosas a partir da espátula metálica. 03–divisão medial do ramo dorsal do nervo ulnar completamente dissecado (seta dupla preenchida) e em anastomose no ponto médio entre o nervo ulnar (08), o ramo profundo do nervo ulnar (09) e o ramo superficial do nervo ulnar (a variante anastomose de Kaplan); 06–osso pisiforme; 07–músculo abdutor do dedo mínimo; 10–nervo digital palmar comum; 11–nervo digital palmar próprio; 12–ramo motor para o músculo palmar curto; e 13–face profunda do músculo palmar curto, parcialmente retraído de sua origem ou inserção distal; M–medial e D–distal.

**Fig. 3:** Sulco no osso pisiforme (6) representado pela linha preta descontínua no sulco e na face medial e anterior deste osso; 03–anastomose tipo Kaplan (seta dupla preenchida); 08–nervo ulnar; 13–face profunda do músculo palmar curto, parcialmente retraída de sua origem ou inserção proximal; 14–ramo profundo da artéria ulnar; asterisco (*) – instrumento cirúrgico puxando a artéria ulnar a partir de sua adventícia; M – medial e D – distal.

## Discussão

A anastomose de Kaplan constitui uma comunicação incomum entre o ramo digital próprio do dedo mínimo (medial) e o ramo dorsal do nervo ulnar 4 5 6 7 e apresenta, devido à sua localização superficial, implicações clínicas e cirúrgicas significativas, inclusive iatrogênicas. 8 No entanto, existem variações desta anastomose, como a conexão atípica do ramo dorsal ao ramo profundo do nervo ulnar conforme descrito por Ghabriel e Makar. 9 Neste estudo, a anastomose ocorreu entre a divisão medial do ramo dorsal do nervo ulnar no ponto médio entre as origens dos ramos superficial e profundo, achado que difere daqueles de Paraskevas et al. 6 e Torre et al., 7 uma vez que ambos estudos relataram anastomose do ramo dorsal do nervo ulnar ao nervo ulnar proximal às emissões dos ramos superficial e profundo. Além disso, a divisão medial anastomótica não suprimiu o aspecto cutâneo da margem medial da mão em direção à face dorsal, o que difere de trabalhos anteriores. Hankins e Flemming 4 propuseram uma classificação, em seis tipos, para as variações da anastomose de Kaplan quanto à conexão e/ou comunicação distal do ramo dorsal do nervo ulnar. No entanto, o presente estudo não se enquadra nessa classificação, porque não houve previsão, na referida classificação, de uma comunicação da divisão medial do ramo dorsal no ponto médio entre os ramos volares do nervo ulnar (superficial e profundo) e porque a divisão medial se relaciona exclusivamente à anastomose. Os achados desses autores evidenciaram um sulco curto no osso pisiforme para acomodação do ramo do nervo variante, que também observamos, porém, o sulco era de maior extensão e profundidade ( Fig. 3 ).
